# Progress in Cancer

**Published:** 1900-11-17

**Authors:** 


### PROGRESS IN CANCER.

**Ætiology.**—Behla[1] has constructed a map from the extensive literature on the subject showing the relative frequency of cancer in various parts of the world. Though necessarily incomplete, it brings to notice several points of interest

Cancer is more prevalent in the middle and southern districts; in the northern parts, in Greenland and Faroe Islands, it is rare or unknown. In Greece and Turkey, however, it is rare. It is rare or unknown in Northern Asia, Arabia, Syria, and Persia; in China less than in Europe; in India rare; in Further India more common; in Japan as in Europe; very little in Africa, but more frequent in Algiers and Madeira; yet in Abyssinia, Tunis, Egypt, and South Africa almost unknown. In Australia cancer shares with tuberculosis and enteritis the first place as the cause of death. In Canada and in South California it is rare; while in the United States it is more frequent, particularly in the large towns; in the tropical, sub-tropical districts, West Indies, Guiana and Brazil, and South generally, rare; in Mexico, Puebla, Ecuador, and Peru commoner.

G. Melmoth Scott,[2] by analysis of statistics of cancer of the low-lying Unions of Chelmsford and Maldon, in East Essex, shows that these areas do not comply with Havilland's theory, viz., that cancer mortality is greatest where rivers, starting from soft marshes or easily disintegrated rock, fall into sheltered valleys, where floods are frequent and the water is charged with alluvial matter, and that the districts characterised by the Tertiary and more recent clays and other retentive soils have a very high mortality from cancer.

In these districts the cancer death-rate bears no evident relation to the variations in the physical features of the country.

(1) Cancer is less common within the county of Essex with its clay soil and numerous estuaries, than it is in England as a whole.

(2) Although the Unions of Chelmsford and Maldon contain an excessive proportion of marshy land and estuary, yet their death-rate is below that of the county.

(3) Those parts of the two Unions which include the greatest extent of muddy foreshore, creek, and "saltings" have a mortality below that of the two Unions taken together.

(4) The disease is not especially prevalent in those places which are situated on the banks of the fresh-water rivers.

A committee set apart to consider the cancer statistics of Worcestershire, &c., publish their conclusions[3] that—

(1) Certain more or less well-defined areas exist in which mortality from cancer is markedly above, and others in which it is markedly below, the average of England and Wales.

(2) Age and sex incidence only account for a small proportion of this variation.

(3) Cancer death-rate is probably under-estimated.

(4) Contamination of soil or subsoil, with decomposing organic matter, is probably a factor in the production of a high death-rate.

(5) Damp, ill-drained, water-logged soil is more often associated with high death-rate than dry, well-drained soil.

(6) Cancer occurs with marked frequency in certain groups of houses, and second and third cases occur in the same house with greater frequency than could be accounted for by mere coincidence.

(7) Cancer occurs more frequently in old than in new houses.

(8) Some evidence suggests that in some cases cancer may be possibly transmitted from one person to another in constant close association.

**Diagnosis.**—Bryant,[1] writing on cysts of the breast, brings forward the following important points:—

(1) Simple cysts of the breast are far more common than is generally believed.

(2) They are chiefly found in women during the same period of life as that in which cancer is met with.

(3) That they are mostly quite amenable to local treatment, without removal of breast.

(4) There is no reason to believe that women who have them are more prone to cancer than those who have not.

Out of 170 cases of his where the diagnosis was verified by operation, 74·1 per cent. were cancerous, while 25·8 per cent. were cystic. This shows, he says, that out of every four cases simulating cancer one proves to be simple cyst, and that simple cyst is therefore more common than is generally supposed. He finds that 77·5 per cent. of simple cysts are in women over forty years of age. He urges these points in diagnosis:—

If the tumour be found to vary in hardness from time to time suspicion that it may be a cyst can be entertained. Cysts may give rise to a discharge from the nipple, of which there may be some retraction in rare cases. The skin may be tense over a cyst, but is not dimpled. If the pressure of the tumour produce a clear discharge from the nipple, cyst may be suggested; but if the discharge be brown or blood-stained, an intra-cystic growth is probable (these he includes under "Cancer"); if pure blood, then soft, solid, malignant growth should be feared. If no discharge cyst is not negatived. Whatever the tumour may be, an exploratory incision is called for, and expectant treatment in no cases advisable.

Trévinot[5] calls attention to the occurrence of endothelioma of bone, which, he says, occurs more frequently than many pathologists believe.

It attacks the spongy tissue and usually the extremities of long bones (though not the articulation), flat bones, cranium, and jaw. The growth is encapsulated. The section is deep red, resembling muscle. A distinguishing feature is the absence of osseous lamellæ.

It may occur at any age and indifferently in either sex. It develops painlessly, so that in many cases spontaneous fracture was the first symptom noticed; no enlarged superficial veins as in sarcoma. The patient becomes weak and cachectic, and succumbs in from 6 to 19 months, with secondary deposits in other bones, lungs, or pleuræ.

The strongly marked pulsation met with in a third of the cases should not be mistaken for aneurysm of bone. This latter, he holds, is a synonym for various forms of growth, and does not exist as aneurysm.

For treatment he suggests resection in early cases and amputation in later.

[1] Brit. Med. Jour., March 24, 1900. [2] Lancet, Aug. 25, 1900. [3] Birmingham Med. Review, July, 1900. [4] Lancet, April 28, 1900. [5] Brit. Med., Jour. Aug. 4, 1900.

